# Determinants of influenza vaccine uptake and willingness to be vaccinated by pharmacists among the active adult population in Hungary: a cross-sectional exploratory study

**DOI:** 10.1186/s12889-021-10572-8

**Published:** 2021-03-17

**Authors:** Githa Fungie Galistiani, Mária Matuz, Nikolett Matuszka, Péter Doró, Krisztina Schváb, Zsófia Engi, Ria Benkő

**Affiliations:** 1 Department of Clinical Pharmacy, Faculty of Pharmacy, University of Szeged, Szikra utca 8, Szeged, 6725 Hungary; 2 Faculty of Pharmacy, Universitas Muhammadiyah Purwokerto, Jalan KH. Ahmad Dahlan, PO BOX 202, Purwokerto, 53182 Indonesia

**Keywords:** Influenza vaccine, Vaccine uptake, Determinants, Adult, Pharmacists

## Abstract

**Background:**

Many studies have addressed influenza vaccine uptake in risk-group populations (e.g. the elderly). However, it is also necessary to assess influenza vaccine uptake in the active adult population, since they are considered to be a high-transmitter group. In several countries pharmacists are involved in adult vaccination in order to increase uptake. This study therefore aimed to investigate the determinants of influenza vaccination uptake and examine the willingness to be vaccinated by pharmacists.

**Methods:**

A cross-sectional study was conducted among Hungarian adults using a self-administered online questionnaire distributed via social media (Facebook). The questionnaire included five domains: demographics, vaccine uptake, factors that motivated or discouraged vaccination, knowledge and willingness of participants to accept pharmacists as influenza vaccine administrators. Descriptive statistics were applied and logistic regression was conducted to assess the possible determinants of vaccination uptake.

**Results:**

Data from 1631 participants who completed the questionnaires were analysed. Almost 58% of respondents (944/1631) had occupational and/or health risk factors for influenza. Just over one-tenth (12.3%;200/1631) of participants were vaccinated during the 2017/18 influenza season, 15.4% (145/944) of whom had a risk factor for influenza. Approximately half of the participants (47.4%) believed that influenza vaccination can cause flu, and just over half of them (51.6%), were not knowledgeable about the safety of influenza vaccine ingredients. Logistic regression found that age, sex, health risk factor and knowledge on influenza/influenza vaccination were associated with influenza vaccination uptake (*p* < 0.05). The most frequently cited reason for having an influenza vaccination was self-protection (95.0%). The most common reason given for refusing the influenza vaccine was that the respondent stated they rarely had an infectious disease (67.7%). The number of participants who were willing to be vaccinated by pharmacists was two-times higher than the number of participants who were actually vaccinated during the 2017/18 influenza season.

**Conclusion:**

Influenza vaccine uptake in the active adult population is low in Hungary. Public awareness and knowledge about influenza vaccination and influenza disease should be increased. The results also suggest a need to extend the role played by pharmacists in Hungary.

## Background

Influenza is a highly infectious viral disease that spreads around the world in annual outbreaks, resulting in between 3 and 5 million cases of severe illness and 290,000–650,000 deaths [1, 2]. One study ranked influenza as the infectious disease with the highest impact on population health in Europe [3].

The most effective way for individuals to avoid this disease is to have an influenza vaccination each year [1, 4]. Influenza vaccination has been recommended by the WHO for some specific populations (e.g. pregnant women and the elderly) [1]. Despite the well-recognised target population for seasonal influenza vaccination, there is some evidence suggesting that vaccination should be also prioritised among those with the highest number of social contacts, i.e. schoolchildren and active adults, to avoid transmission of infections and large outbreaks [5, 6]. Additionally, protective immune response after vaccination may develop in higher rate in the young ones, compared to elderly with immunosenescence [7].

Factors relating to influenza vaccine uptake have previously been investigated mainly in the Western European countries and the USA [8, 9]; a limited number of studies have been performed in Central and Eastern European countries [8–10]. In a multi-site study from eleven European countries, various factors (e.g. socio-economics factors, gender, size of household, educational level and household income) were identified as potential determinants of influenza vaccine uptake, but no data was reported for Hungary [10]. Therefore, there is a need to assess and understand factors that may influence influenza vaccination uptake in Hungary. Beside the lack of knowledge on associated factors of influenza uptake in Hungary, the other motivation of this research was that no other studies focused specifically on the active adult population, which may play crucial role in flu epidemic development.

A review article summarised the strategies that have been applied in an attempt to achieve higher coverage rates for influenza vaccination [11]. As access to influenza vaccination is an important challenge in many countries, one of the recommended strategies was the involvement of community pharmacists as influenza vaccine administrators, due to their better access to patients and more convenient opening time. Pharmacy-based vaccination services have been gradually developing since the end of the twentieth century. These services were first established in Argentina, South-Africa, USA and Australia, but have since expanded to some European countries (Denmark, Ireland, Portugal, Switzerland and the UK) and also to some countries outside of Europe (Canada, Philippines) [12–14]. A systematic review and meta-analysis showed that the involvement of pharmacists in vaccination programmes, whether as educators, facilitators or administrators of vaccines, resulted in increased vaccination rates [15]. Accordingly, the International Federation of Pharmacists (FIP) has a strong commitment to improve vaccination coverage through pharmacists and actively advocate pharmacy vaccination for more than a decade [14, 16]. Despite the well-recognised benefits of the involvement of pharmacists, no pharmacy-based vaccination services currently exist in Central or Eastern Europe and it is important to observe whether the patients willing to be vaccinated by pharmacists in Hungary. As access to influenza vaccination may be challenging for Hungarian adults as well, there is potential to improve access and vaccination uptake by enabling pharmacists to give flu vaccinations.

The main objective of the present study was to investigate the determinants of influenza vaccination uptake in the active adult population in Hungary and secondly to explore participants’ willingness to accept pharmacists as influenza vaccine administrators.

## Methods

### Study design and setting

The study was an observational cross-sectional study carried out in Hungary between March and July 2018. The self-administered questionnaire was distributed via social media (*Facebook*). *Facebook* is the most popular social media used in Hungary and majority (80%) of the users belong to the 20–59 years age group [17]. The questionnaire was constructed on *Google docs* and the link was shared to public via various Hungarian *Facebook Groups (N = 35)*. In order to achieve a neutral sampling, we targeted participants based on various leisure time activities (i.e. different newspaper readers, fishermen, bee keepers, cooking groups, etc.). First, we contacted the group administrators to put the link on the open page and sent them reminders for 2–3 times.

### Participants

Everyone who lives in Hungary, understands Hungarian and has a *Facebook* account was eligible to voluntarily take part in this study. No financial or other incentives were applied. During the analysis, however, we focused on the active adult population, aged 20 to 59 years.

### Sample size

We adopted the sample size calculation written by Lemeshow et al. and published by the World Health Organisation [18]. We assumed that each of the main outcome measures has a prevalence between 5 and 95% (not very rare or very frequent). After we targeted the highest minimal sample size (*N* = 384) that could be required, with a precision estimate of ±5% and the type I error (alpha) of 5% at 95% confidence level.

### Questionnaire

The survey instrument was a questionnaire. The questions included general characteristics (e.g. age, sex, risk factors for influenza based on The Annual Vaccine Guideline of the Hungarian Ministry of Health [19]), uptake of the seasonal influenza vaccine during the 2017/18 influenza season, factors motivating or discouraging uptake of the vaccine (see complete list of questions in Table 3), participants’ knowledge in relation to influenza and influenza vaccination (see complete list of questions in Table 5) and the willingness of the participants to receive an influenza vaccination from their community pharmacist. Questions on potential determinants and knowledge items were based on published studies [20–23] and own ideas of the study team. The study team discussed potential questions at several rounds, and included questions after consensus. Then the questionnaire was piloted with a sample of ten individuals to ensure the clarity of the questions.

Binary questions were asked about both seasonal influenza vaccination uptake and the willingness of participants to receive an influenza vaccination from a pharmacist. Multiple choice questions were used to gain information relating to factors motivating or discouraging influenza vaccination uptake.

The knowledge of participants relating to influenza/influenza vaccination was measured using a set of 17 questions. For each question, there were three possible answers: ‛yes’, ‛no’ or ‛don’t know’. One point was assigned for each correct answer, zero points were given for the ‛don’t know’ answer and one point was subtracted for giving the wrong answer. Finally, the total was calculated, with a range from − 17 to + 17, then calculated as the percentage of the total achievable points. For this knowledge section, the answers of participants who had more than five missing answers were excluded from the analysis.

### Data analysis

Descriptive, bivariate and multivariate statistical analyses were applied to describe all survey items. Descriptive statistics, including means, standard deviations and percentages were used to describe all variables. Bivariate analyses, such as Pearson’s chi-square test and Fisher’s exact test, were used to compare categorical variables. Logistic regression was conducted to assess the potential associated factors of influenza vaccination uptake and adjusted odds ratios were reported. The level of statistical significance was set at *p* < 0.05. All statistical analyses were performed using R software (R version 3.6.1).

## Results

**Fig. 1:**
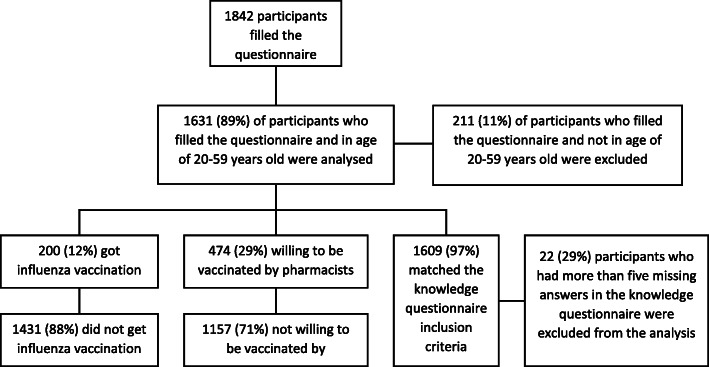
Flow chart of participants in the study

In total, 1842 questionnaires were filled. Of these, 1631 questionnaires were analysed and 211 were excluded since those were filled by participants who were not in the active adult (20–59 years old) population (Fig. 1). The mean age of participants was 33.7 years (SD = 10.7; CI 95% 33.2–34.2), while 944 participants (57.9%; CI 95% 55.5–60.3) had occupational and/or health risk factors for influenza. Just over one-tenth (12.3%; CI 95% 10.8–13.9) of participants had received an influenza vaccination during the previous influenza season, and 15.4% (145/944; CI 95% 13.2–17.8) of those had a risk factor for influenza. The general characteristics of the participants are presented in Table 1.

**Table 1:** Bivariate analysis of participants’ general characteristics and influenza vaccination uptake during the 2017/18 influenza season

Variable	Vaccination status			Statistical test	***p***-value
	Yes (*n* = 200)	No (*n* = 1431)	Total		
	Number (Row %)	Number (Row %)	Number (Col.%)		
**Age** (years)					
(mean ± SD)	37.5 ± 11.2	33.2 ± 10.5	33.7 ± 10.7	Welch’s two Sample t-test	< 0.001
**Sex**					
Male	50 (16.1)	260 (83.9)	310 (19.0)	Pearson’s Chi-square test	0.0214
Female	150 (11.4)	1170 (88.6)	1320 (80.9)		
*N/A*	0 (00.0)	1 (100)	1 (00.0)		
**Type of residence**					
Village	33 (13.3)	225 (86.8)	258 (15.8)	Pearson’s Chi-square test	0.7587
City	166 (12.1)	1205 (87.9)	1371 (84.1)		
*N/A*	1(0.5)	1 (0.5)	2 (0.1)		
**Educational level**					
Primary	4 (15.4)	22 (84.6)	26 (1.6)	Fisher’s exact test for count data	0.0146
Secondary	87 (10.1)	772 (89.9)	859 (52.7)		
Tertiary	109 (14.7)	635 (85.4)	744 (45.6)		
*N/A*	0 (0.0)	2 (100)	2 (0.1)		
**Occupational risk factor** ^**a**^					
Yes	88(17.2)	424(82.8)	512(31.4)	Pearson’s Chi-square test	< 0.001
No	112(10.1)	1002(89.9)	1114(68.3)		
*N/A*	0(0.0)	5(100)	5(0.3)		
**Health risk factor** ^**b**^					
Yes	95(15.1)	536(84.9)	631(38.7)	Pearson’s Chi-square test	0.0063
No	105(10.5)	895(89.5)	1000(61.3)		
**Knowledge** (*N* = 1609)					
(mean ± SD)	85.4 ± 9.7	70.7 ± 15.1	72.5 ± 15.3	Welch’s two Sample t-test	< 0.001

^a^Occupational risk factors include participants who have at least one of the following statuses: students in the health care field; work in health care services; social institution/long care term facility; nursery school/kindergarten; livestock or animal transfer (swine, poultry, horse); poultry processing or abattoir; work with immigrants/foreign people

^b^Health risk factors include participants who had at least one of the following conditions in the previous year: heart failure; coronary artery disease; chronic pulmonary disease; immune disease; taking immunosuppressive drugs; inflammatory bowel disease; chronic liver disease; chronic kidney disease; pregnancy/planning pregnancy; disabled (physically); smoker

### Demographics relating to vaccination uptake among participants

Overall, there were significant differences in age, sex, educational level, occupational risk factor, health risk factor and knowledge of vaccinated versus unvaccinated participants (*p* < 0.05) in the bivariate analysis. Participants’ type of residence was the only variable that showed no significant difference between vaccinated and unvaccinated participants (Table 1). Furthermore, logistic regression showed that age, sex, health risk factor and knowledge about influenza were associated with influenza vaccination uptake (Table 2).

**Table 2:** Logistic regression analysis to identify associated factors for influenza vaccination uptake (*n* = 1602)

	OR	95% CI	***p***-value
**Age**	1.028	1.012–1.044	0.001
**Sex** (male)	1.838	1.217–2.774	0.004
**Occupational risk factor**	1.211	0.838–1.751	0.309
**Health risk factor**	2.070	1.472–2.910	0.000
**Educational level –** Primary (reference)	–	–	–
Secondary	0.568	0.149–2.171	0.408
Tertiary	0.585	0.153–2.241	0.434
**Knowledge**	1.096	1.078–1.114	0.000

### Factors which motivated or discouraged vaccination uptake

The reasons for obtaining or not obtaining the influenza vaccination are summarised in Table 3. The most commonly cited reasons for having the vaccination were ‛self-protection’ (95.0%), ‛to protect those with risk factors around’ (61.0%) and ‛consider influenza as severe disease’ (52.5%). The most cited reasons for not having the vaccination were ‛I rarely get infectious diseases’ (67.7%), followed by ‛I do not have a risk factor’ (48.9%) and ‛nobody has risk factor around’ (41.0%). In total, 700 (48.9%) unvaccinated participants selected ‛I do not have a risk factor’ as their reason for not having the vaccination; however, we discovered that in reality just over half of them (353/700) had at least one risk factor for influenza.

**Table 3:** Participants’ cited reasons for their vaccination status^a^

Reasons for being …	Number (%)
**Vaccinated**, *n* = 200 (100%)	
To protect myself from the flu and its complications	190(95.0)
To protect those with risk factors around me	122(61.0)
I consider flu a severe disease	105(52.5)
I belong to a risk group, I am prone to infections/diseases	69(34.5)
I had severe influenza previously	31(15.5)
Death due to influenza complications around me (in my neighbourhood)	7(3.5)
**Unvaccinated**, *n* = 1431 (100%)	
Because I rarely get infectious diseases (including influenza)	968(67.7)
Because I do not have a risk factor	700(48.9)
Because nobody has a risk factor around me	587(41.0)
Because I prefer alternative therapies (e.g. natural medicine)	511(35.7)
I consider influenza a minor disease	463(32.4)
Because I am afraid of the side effects	451(31.5)
Because of the contradictory opinions on flu vaccine	411(28.7)
I consider the vaccine ineffective	380(26.6)
Because I prefer medical therapy	361(25.2)
Previous bad experience with flu vaccine among family members/acquaintance	341(23.8)
Because I forgot and missed it	263(18.4)
Because I am afraid of the needles	172(12.0)
I received the flu vaccine previously, but it was ineffective because I got the flu	156(10.9)
Influenza vaccine is contraindicated to me	93(6.5)
I received the flu vaccine previously, but I experienced major/serious side effects	85(5.9)

^a^Participants’ could give more than one reason

Table 4 shows the role played by different sources of advice or opinions when it came to participants’ vaccination status. Most participants stated that they were not influenced (indifferent or not influenced categories) by any external opinions with regard to their influenza vaccination uptake. Approximately one-third of vaccinated participants stated that their decision had been influenced by a recommendation from a specialist doctor, a GP, another healthcare worker or a family member.

**Table 4:** The role played by different sources of recommendations/opinions on individuals’ vaccination uptake decision

Source of recommendation or opinion	To have the influenza vaccine *n* = 200 (100%)	To not have the influenza vaccine *n* = 1431 (100%)
	Number (%)	Number (%)
**Specialist**		
Influenced	75(37.5)	234(16.4)
Indifferent	19(9.5)	256(17.9)
Not influenced	84(42.0)	841(58.8)
*N/A*	22(11.0)	100(7.0)
**Family member**		
Influenced	70(35.0)	368(25.7)
Indifferent	32(16.0)	280(19.6)
Not influenced	77(38.5)	701(49.0)
*N/A*	21(10.5)	82(5.7)
**General practitioner**		
Influenced	68(34.0)	187(13.1)
Indifferent	27(13.5)	277(19.4)
Not influenced	85(42.5)	861(60.2)
*N/A*	20(10.0)	106(7.4)
**Other healthcare worker**		
Influenced	62(31.0)	301(21.0)
Indifferent	26(13.0)	266(18.6)
Not influenced	89(44.5)	783(54.7)
*N/A*	23(11.5)	81(5.7)
**Pharmacist**		
Influenced	28(14.0)	173(12.1)
Indifferent	28(14.0)	279(19.5)
Not influenced	112(56.0)	870(60.8)
*N/A*	32(16.0)	109(7.6)
**Media (internet/television/radio)**		
Influenced	12(6.0)	109(7.6)
Indifferent	30(15.0)	330(23.1)
Not influenced	124(62.0)	888(62.1)
*N/A*	34(17.0)	104(7.3)

### Knowledge of influenza vaccination/influenza disease

The participants’ knowledge in response to certain questions is summarised in Table 5. Most participants (93.6%) knew that influenza is an infectious disease. On the other hand, approximately half of the participants (47.4%) believed that influenza vaccination can cause flu, and just over half of them (51.6%), calculated from the sum of ‘wrong’ and ‘unknown’ answers) were not knowledgeable about the safety of influenza vaccine ingredients (Table 5).

**Table 5:** Participants’ knowledge about influenza and influenza vaccination

Question	Correct answer	Answer	Vaccinated *n* = 200 (100%)	Unvaccinated *n* = 1431 (100%)	Total *n* = 1631 (100%)
			Number (%)	Number (%)	Number (%)
Influenza is an infectious disease	True	Correct	194(97.0)	1333(93.2)	1527(93.6)
		Wrong	4(2.0)	57(4.0)	61(3.7)
		Do not know	0(0.0)	30(2.1)	30(1.8)
		*NA*	2(1.0)	11(0.8)	13(0.8)
Influenza vaccination is recommended annually for the risk groups	True	Correct	194(97.0)	1122(78.4)	1316(80.7)
		Wrong	0(0.0)	87(6.1)	87(5.3)
		Do not know	3(1.5)	205(14.3)	208(12.8)
		*NA*	3(1.5)	17(1.2)	20(1.2)
Influenza vaccination is highly/specially recommended for the elderly	True	Correct	189(94.5)	988(69.0)	1282(78.6)
		Wrong	1(0.5)	148(10.3)	119(7.3)
		Do not know	6(3.0)	280(19.6)	209(12.8)
		*NA*	4(2.0)	15(1.1)	21(1.3)
Influenza vaccination is highly/specially recommended for those with chronic diseases	True	Correct	185(92.5)	1093(76.4)	1173(71.9)
		Wrong	4(2.0)	118(8.3)	152(9.3)
		Do not know	9(4.5)	203(14.2)	289(17.7)
		*NA*	2(1.0)	17(1.2)	17(1.0)
Elderly and those with certain chronic diseases can get the flu vaccination for free	True	Correct	183(91.5)	1009(70.5)	1192(73.1)
		Wrong	4(2.0)	48(3.4)	52(3.2)
		Do not know	11(5.5)	360(25.2)	371(22.8)
		*NA*	2(1.0)	14(1.0)	16(1.0)
Influenza is a synonym for common cold	False	Correct	179(89.5)	1239(86.6)	1418(86.9)
		Wrong	9(4.5)	106(7.4)	115(7.1)
		Do not know	7(3.5)	73(5.1)	80(4.9)
		*NA*	5(2.5)	13(0.9)	18(1.1)
In case of fever the vaccination should be postponed	True	Correct	177(88.5)	1176(82.2)	1353(83.0)
		Wrong	7(3.5)	41(2.9)	48(2.9)
		Do not know	13(6.5)	199(13.9)	212(13.0)
		*NA*	3(1.5)	15(1.1)	18(1.1)
The best method to prevent influenza is the influenza vaccination	True	Correct	172(86.0)	619(43.3)	791(48.5)
		Wrong	12(6.0)	453(31.7)	465(28.5)
		Do not know	13(6.5)	339(23.7)	352(21.6)
		*NA*	3(1.5)	20(1.4)	23(1.4)
Time to onset of action is 2 weeks for influenza vaccination in case of adults	True	Correct	163(81.5)	841(58.8)	1004(61.6)
		Wrong	6(3.0)	68(4.8)	74(4.5)
		Do not know	29(14.5)	501(35.0)	530(32.5)
		*NA*	2(1.0)	21(1.5)	23(1.4)
Influenza vaccine contains safe ingredients	True	Correct	159(79.5)	606(42.4)	765(46.9)
		Wrong	8(4.0)	258(18.0)	266(16.3)
		Do not know	31(15.5)	544(38.0)	575(35.3)
		*NA*	2(1.0)	23(1.6)	25(1.5)
Influenza can be prevented by high dose vitamin C (min. 500 mg daily) instead of vaccination	False	Correct	156(78.0)	662(46.3)	818(50.2)
		Wrong	12(6.0)	407(28.4)	419(25.7)
		Do not know	30(15.0)	344(24.0)	374(22.9)
		*NA*	2(1.0)	18(1.3)	20(1.2)
Antibiotics work against influenza	False	Correct	155(77.5)	953(66.6)	1108(67.9)
		Wrong	24(12.0)	321(22.4)	345(21.2)
		Do not know	18(9.0)	144(10.1)	162(9.9)
		*NA*	3(1.5)	13(0.9)	16(1.0)
The flu vaccination can weaken the immune system	False	Correct	151(75.5)	652(45.6)	803(49.2)
		Wrong	24(12.0)	439(30.7)	463(28.4)
		Do not know	22(11.0)	320(22.4)	342(21.0)
		*NA*	3(1.5)	20(1.4)	23(1.4)
Influenza can be prevented by herbs (e.g. honey, ginger tea) instead of vaccination	False	Correct	143(71.5)	672(47.0)	815(50.0)
		Wrong	13(6.5)	374(26.1)	387(23.7)
		Do not know	41(20.5)	368(25.7)	409(25.1)
		*NA*	3(1.5)	17(1.2)	20(1.2)
You should not take influenza vaccination if you have already got influenza	False	Correct	117(58.5)	625(43.7)	742(45.5)
		Wrong	49(24.5)	421(29.4)	470(28.8)
		Do not know	32(16.0)	370(25.9)	402(24.7)
		*NA*	2(1.0)	15(1.0)	17(1.0)
The flu vaccination can cause flu	False	Correct	109(54.5)	390(27.3)	499(30.6)
		Wrong	62(31.0)	711(49.7)	773(47.4)
		Do not know	27(13.5)	308(21.5)	335(20.5)
		*NA*	2(1.0)	22(1.5)	24(1.5)
In case of egg allergy, the flu vaccination can be taken	True	Correct	58(29.0)	323(22.6)	381(23.4)
		Wrong	61(30.5)	264(18.5)	325(19.9)
		Do not know	79(39.5)	822(57.4)	901(55.2)
		*NA*	2(1.0)	22(1.5)	24(1.5)

In total, only 30.6% of all participants gave a correct answer to the statement ‛flu vaccine can cause influenza disease’; however, the vaccinated group showed better knowledge compared with the knowledge of the unvaccinated group (54.5% vs 27.3%) (Table 5). Moreover, vaccinated participants scored higher for each knowledge question in comparison with the scores of non-vaccinated participants. This higher level of knowledge was identified as one of the factors associated with influenza vaccine uptake (*p* < 0.05) (Table 2).

There were large differences in the level of knowledge between vaccinated and unvaccinated participants with regard to assumptions that influenza vaccination is the best method to prevent influenza (12.5% vs 55.4%), the safety of vaccine ingredients (19.5% vs 56.1%) and whether the influenza vaccine can cause influenza disease (44.5% vs 71.2%) (These numbers were calculated from the sum of ‘wrong’ and ‘unknown’ answers in Table 5).

### Willingness to accept pharmacists as influenza vaccine administrators

Overall, almost one-third (29.1%; CI 95% 26.9–31.3) of all participants would accept an influenza vaccination from a pharmacist. Table 6 shows that the willingness to accept pharmacists as vaccine administrators was significantly higher among participants who had been vaccinated during the last influenza season (*p* < 0.05). Similarly, the mean knowledge score of participants who were willing to get their influenza vaccination at a pharmacy was significantly higher compared with the knowledge of those who said they would refuse a pharmacy-based service (*p* < 0.05). The number of participants who were willing to be vaccinated by pharmacists (*n* = 474) was two times higher than the number of participants who were actually vaccinated during the 2017/18 influenza season (*n* = 200).

**Table 6:** Bivariate analysis of general characteristics and participants’ willingness to be vaccinated by a pharmacist

Variable	Willingness to be vaccinated by a pharmacist		Statistical test	***p-value***
	Yes *n* = 474 (100%)	No *n* = 1157 (100%)		
	Number (%)	Number (%)		
**Age** (years)				
(mean ± SD)	32.5 ± 10.8	34.2 ± 10.6	Welch’s two sample t-test	0.0029
**Sex**				
Male	132(42.6)	178(57.4)	Pearson’s Chi-square test	< 0.001
Female	341(25.8)	979(74.2)		
*N/A*	1(100)	0(0.0)		
**Type of residence**				
Village	61(23.6)	197(76.4)	Pearson’s Chi-square test	0.0375
City	412(30.0)	959(70.0)		
*N/A*	1(50.0)	1(50.0)		
**Education level**				
Primary	14(53.9)	12(46.1)	Pearson’s Chi-square test	0.0192
Secondary	245(28.5)	614(71.5)		
Tertiary	214(28.8)	530(71.2)		
*N/A*	1(50.0)	1(50.0)		
**Occupational risk** ^a^				
Yes	122(23.8)	390 (76.2)	Pearson’s Chi-square test	0.0014
No	352(31.6)	762(68.4)		
*N/A*	0(0.0)	5(100)		
**Health conditions risk** ^b^				
Yes	179(28.4)	452(71.6)	Pearson’s Chi-square test	0.6238
No	295(29.5)	705 (70.5)		
**Vaccinated**				
Yes	116(58.0)	84(42.0)	Pearson’s Chi-square test	< 0.001
No	358(25.0)	1073(75.0)		
**Knowledge** (*n* = 1609)				
(mean ± SD)	79.3 ± 12.4	69.8 ± 15.5	Welch’s two sample t-test	< 0.001

^a^Occupational risk factors include participants who have at least one of the following statuses: students in the healthcare field; work in health care services; social institution/long care term facility; nursery school/kindergarten; livestock or animal transfer (swine, poultry, horse); poultry processing or abattoir; work with immigrants/foreign people

^b^Health risk factors include participants who had at least one of following conditions in the previous year: heart failure; coronary artery disease; chronic pulmonary disease; immune disease; taking immunosuppressive drugs; inflammatory bowel disease; chronic liver disease; chronic kidney disease; pregnancy/planning pregnancy; disabled (physically); smoker

## Discussion

There are limited data available relating to influenza vaccine uptake patterns in Central and Eastern Europe. The technical report of the ECDC on seasonal influenza coverage rate [24] showed that in Hungary, vaccination rate among the elderly (above 60 years) was 21.9% in 2017/2018, which is far from the target of 75%. In the present study focusing on only active adults, the influenza vaccination uptake of respondents was low, 12.3%. A similarly low level (9.5%) of vaccination coverage was reported from Poland (considering the whole population), and generally influenza vaccination uptake was suboptimal across Europe [10]. More than half (944/1631) of participants in the present study had occupational and/or health risk factors, and only 15.4% of them had been vaccinated against influenza. Recent studies have reported that vaccination rates among adults aged 16 to 65 years old who had a risk factor were higher, at between 29.8 and 49.2% in Australia and between 45.7 and 49.4% in England [25, 26].

Our findings showed that approximately one in two participants believed that the influenza vaccine can cause influenza (47.4%) and half of them were not knowledgeable about the safety of influenza vaccine ingredients (51.6%). These factors might have influenced these participants’ decisions not to have an influenza vaccination during the 2017/18 influenza season.

### Demographics relating to vaccination uptake among participants

With regard to the demographic factors associated with influenza vaccine uptake, some of our findings are similar to previously published findings. In the present study, older age was associated with influenza vaccination uptake (Table 2). A similar finding was reported by a systematic review that focused on European and Asian populations [27]. The present study also showed that being male was associated with being vaccinated (Table 2). Additionally, some studies have noted that being female can be a barrier to influenza vaccine uptake [8, 10, 28]. However, the aforementioned systematic review reported that sex was not a consistent predictor of influenza vaccination across different European countries [27].

Another factor associated with influenza vaccination uptake was having a health risk factor. A similar association between health risk factors and vaccine uptake has been reported in some previous studies [20, 28, 29]. However, the earlier systematic review found that occupational health risk factors were not a consistent predictor of influenza vaccination uptake [27].

### Factors that motivated or discouraged vaccination uptake

The most frequently cited reasons for having an influenza vaccination were ‛self-protection’ and ‛to protect those with risk factors around’, which were also noted in other studies [20, 22, 30], followed by ‛consider flu as a severe disease’. These stated reasons imply that vaccinated people are more likely to be aware of the negative impacts of influenza disease. Of note, social responsibility was an important motivating factor for influenza vaccination. In the unvaccinated group, the most frequently stated reasons for not having influenza vaccination were ‛rarely get influenza’, followed by ‛I do not have a risk factor’. Surprisingly, we found that participants who selected ‛I do not have a risk factor’ in fact had at least one existing risk factor. It can be assumed that participants’ perception of risk factors needs to be improved through some type of educational intervention by healthcare professionals. Previous studies have also shown that the low uptake of the influenza vaccine is related to the perceived low risk of the disease [8, 20, 28, 30–32].

More than one-third of vaccinated participants reported that healthcare workers or a family member influenced their decision to have the influenza vaccine. Previous studies have also reported that a recommendation or opinion from healthcare workers or family members is a factor that influences whether someone has an influenza vaccination [8, 20, 30, 33]. Interestingly, some of participants stated that they were influenced by healthcare workers (specialist, GP, pharmacist and other HCWs) not to take the influenza vaccine; the reason for this are unclear. It is possible that HCWs may have their own personal beliefs regarding to influenza and/or influenza vaccination. A systematic review showed that HCW’s personal beliefs may act as barriers to vaccine uptake, including concerns about side effects, scepticism about vaccine effectiveness and the belief that influenza is not a serious illness [34].

However, most participants in the present study stated that their decision was not influenced by any external source. It can be concluded that most participants’ decisions whether to have the influenza vaccination were mainly influenced by their own perceptions about influenza disease and/or the influenza vaccine. A previous systematic review found that perceptions around vaccine efficacy, safety and adverse events were the most influential factors in influenza vaccination uptake [27]. Consequently, educational interventions relating to influenza disease and/or influenza vaccine should be targeted at patients themselves.

### Knowledge about influenza vaccination/influenza disease

This survey found that participants’ level of knowledge around influenza vaccination and influenza disease associated with influenza vaccine uptake. Other studies have also found a higher level of knowledge to be associated with higher vaccination uptake rates [21, 27, 29, 35]. Additionally, previous research has shown that a lack of general knowledge about influenza/influenza vaccination was a barrier to influenza vaccination uptake [8].

Large differences existed in the level of knowledge between vaccinated and unvaccinated groups with regard to assumptions around the best method to prevent influenza, the safety of vaccine ingredients and whether the influenza vaccine can cause influenza disease. Overall, the vaccinated participants were more knowledgeable than the unvaccinated ones in all other question items. These findings provide evidence that the lack of knowledge regarding the effectiveness and safety of the influenza vaccine in the unvaccinated group might influence these participants’ attitudes towards influenza vaccination.

### Willingness to accept pharmacists as influenza vaccine administrators

Previous studies have suggested that pharmacy-provided vaccines may increase the uptake of the influenza vaccine [15, 36, 37]. In Hungary, pharmacy-provided vaccines are not yet available. However, this study found that almost one-third (29.1%) of participants would be willing to receive their influenza vaccine from pharmacists. The participants’ willingness in this regard may indicate that some of them already trust pharmacists to be vaccine administrators.

The results of the statistical analysis showed significant differences in the general characteristics of those who, in principle, said they accepted (the ‛willing group’) and those who, in principle, said they would refuse (the ‛unwilling group’) pharmacists as vaccine administrators. Of the demographic factors, sex, occupational risk factor, level of knowledge and vaccination status were variables having clinical relevance. These findings imply that male participants, participants with an occupational risk factor, participants with a higher level of knowledge about influenza vaccination/influenza disease and those who had been vaccinated against influenza were more willing to be vaccinated by pharmacists.

We observed that the number of participants who were willing to be vaccinated by pharmacists was two-times higher than the number of participants who were actually vaccinated during the 2017/18 influenza season. These findings suggest that influenza vaccination uptake in Hungary might be increased if pharmacists were involved. A number of studies into pharmacy-based influenza vaccination services have been published [12, 15, 38]. Studies have reported that patient satisfaction with pharmacist-administered vaccination was high [39–41]. Being vaccinated by pharmacists would also provide additional educational opportunities. For example, pharmacists can deliver correct information about the safety and quality of the influenza vaccine (e.g. the safety of the ingredients and quality assurance of the product).

### Strengths and limitations

The strength of this study was the large sample size used to assess influenza vaccine uptake, related knowledge, and potential role of pharmacist as vaccine administrators. in Hungary. The findings in this study are, however, subject to some limitations. Data were self-reported by participants who were voluntary recruited via *Facebook* that could induce selection bias and the reported vaccination rates could not be verified by checking participants’ medical records (recall bias). Due to the method, the share of inhabitants below 40 years of age were overrepresented in the study group which leads to slight underestimation of influenza vaccination uptake. Those who are lacking internet access or Facebook account are not represented in the study group. As social media access might be associated with level of influenza vaccine acceptance, either directly or indirectly, this limitation may lead to over- or under-estimation of influenza vaccination coverage. The study of Ahmed et al. from the U.S. showed that users of Facebook or Twitter had higher influenza vaccination uptake, compared to non-users of social media [42]. On the contrary, a strong anti-vaccine content on *Facebook* were detected in some countries [43–45] (note that nowadays *Facebook* is reducing the distribution of misinformation about vaccination and increasing users’ exposure to credible, authoritative information) [46]. In Hungary, the presence of anti-vaccine content might only slightly influence the research findings, since the level of public trust in compulsory vaccinations are above 90% in Hungary and compulsory childhood vaccine uptake is close to 100%, which is outstanding in Europe [46].

Based on the survey method, our results may not represent well the whole Hungarian population. On the other hand, this exploratory study clearly identified problematic areas where educational interventions should focus.

## Conclusions

Influenza vaccine uptake among active adults was low in Hungary. Increased public awareness and improved knowledge about influenza vaccination and/or influenza disease is necessary to achieve higher influenza vaccination uptake rates. Based on the insufficient knowledge of participants concerning the effectiveness and safety of the influenza vaccine, combined with the level of acceptance among participants to obtain an influenza vaccination from a pharmacist, we recommend that both the educational role played by pharmacists should be extended, while vaccine administrator role should be considered and implemented.

## Data Availability

Data will be made available on request from the corresponding author.

## Abbreviations

GP: General practitioner
HCW: Healthcare worker
USA: United States of America
WHO: World Health Organization
